# Effect of deletion of the protein kinase PRKD1 on development of the mouse embryonic heart

**DOI:** 10.1111/joa.14033

**Published:** 2024-02-28

**Authors:** Qazi Waheed‐Ullah, Anna Wilsdon, Aseel Abbad, Sophie Rochette, Frances Bu'Lock, Marc‐Phillip Hitz, Gregor Dombrowsky, Friederike Cuello, J. David Brook, Siobhan Loughna

**Affiliations:** ^1^ School of Life Sciences, Faculty of Medicine and Health Sciences University of Nottingham Nottingham UK; ^2^ East Midlands Congenital Heart Centre University Hospitals of Leicester NHS Trust Leicester UK; ^3^ Institute of Medical Genetics Carl von Ossietzky University Oldenburg Oldenburg Germany; ^4^ Institute of Experimental Pharmacology and Toxicology, Cardiovascular Research Center University Medical Center Hamburg‐Eppendorf Hamburg Germany; ^5^ DZHK (German Center for Cardiovascular Research), partner site Hamburg/Kiel/Lübeck University Medical Center Hamburg‐Eppendorf Hamburg Germany

**Keywords:** congenital heart disease, high‐resolution episcopic microscopy, *Prkd1*, protein kinase, protein kinase D1

## Abstract

Congenital heart disease (CHD) is the most common congenital anomaly, with an overall incidence of approximately 1% in the United Kingdom. Exome sequencing in large CHD cohorts has been performed to provide insights into the genetic aetiology of CHD. This includes a study of 1891 probands by our group in collaboration with others, which identified three novel genes—*CDK13, PRKD1*, and *CHD4*, in patients with syndromic CHD. *PRKD1* encodes a serine/threonine protein kinase, which is important in a variety of fundamental cellular functions. Individuals with a heterozygous mutation in *PRKD1* may have facial dysmorphism, ectodermal dysplasia and may have CHDs such as pulmonary stenosis, atrioventricular septal defects, coarctation of the aorta and bicuspid aortic valve. To obtain a greater appreciation for the role that this essential protein kinase plays in cardiogenesis and CHD, we have analysed a *Prkd1* transgenic mouse model (*Prkd1* ^*em1*^) carrying deletion of exon 2, causing loss of function. High‐resolution episcopic microscopy affords detailed morphological 3D analysis of the developing heart and provides evidence for an essential role of *Prkd1* in both normal cardiac development and CHD. We show that homozygous deletion of *Prkd1* is associated with complex forms of CHD such as atrioventricular septal defects, and bicuspid aortic and pulmonary valves, and is lethal. Even in heterozygotes, cardiac differences occur. However, given that 97% of *Prkd1* heterozygous mice display normal heart development, it is likely that one normal allele is sufficient, with the defects seen most likely to represent sporadic events. Moreover, mRNA and protein expression levels were investigated by RT‐qPCR and western immunoblotting, respectively. A significant reduction in *Prkd1* mRNA levels was seen in homozygotes, but not heterozygotes, compared to WT littermates. While a trend towards lower PRKD1 protein expression was seen in the heterozygotes, the difference was only significant in the homozygotes. There was no compensation by the related *Prkd2* and *Prkd3* at transcript level, as evidenced by RT‐qPCR. Overall, we demonstrate a vital role of *Prkd1* in heart development and the aetiology of CHD.

## Graphical abstract

Congenital heart disease accounts for a third of all congenital defects. The role of protein kinase D1 in cardiogenesis and CHD was studied using a transgenic mouse model (Prkd1em1) carrying deletion of exon 2 (em1), causing loss of function. Morphological 3D analysis by high‐resolution episcopic microscopy has shown a range of CHDs occur in the Prkd1em1 embryonic day E15.5 and the postnatal day (P) 6/7 hearts. Ao, aorta; AS; atrial septum; LA, left atrium; LV, left ventricle; PT, pulmonary trunk; RA, right atrium; RV, right ventricle; VS, ventricular septum.

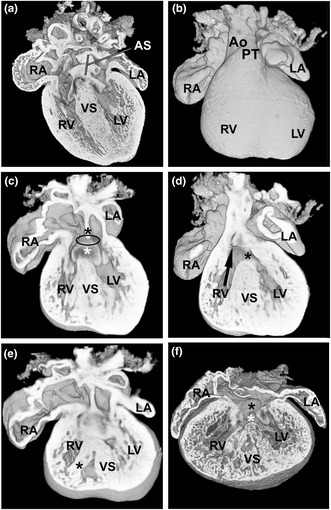

## 1 INTRODUCTION

Congenital heart disease (CHD) is the most common congenital defect in newborns (Kalisch‐Smith et al., 2020; Liu et al., 2019). Genetic causes identified include chromosomal abnormalities, copy number variants and point mutations. Recent studies have used exome sequencing in large CHD cohorts to better understand the genetic contribution to CHD (Homsy et al., 2015; Jin et al., 2017; Richter et al., 2020; Sifrim et al., 2016; Zaidi et al., 2013). 8% of CHD cases were described with de novo variants in over 400 genes (Jin et al., 2017). Variants in genes involved in protein phosphorylation, chromatin modification, cardiac development and neural tube development are significantly over‐represented in syndromic‐CHD patients (Sifrim et al., 2016). Three novel CHD‐causing genes recently identified by our group were *PRKD1, CDK13*, and *CHD4* (Sifrim et al., 2016). Individuals with a variant in *PRKD1* characteristically have facial dysmorphism, ectodermal dysplasia (with dysplastic changes in skin, teeth and nails) and CHD, such as pulmonary stenosis, atrioventricular septal defects, coarctation of the aorta and/or bicuspid aortic valve (Alter et al., 2021; Jin et al., 2017; Sifrim et al., 2016). The mutational spectrum includes both missense and loss of function variants. To date, there are 24 different heterozygous *PRKD1* variants in a total of 31 patients which have been documented in public repositories ClinVar (Landrum et al., 2018) and Decipher (Firth et al., 2009) or in the literature (Alter et al., 2021; Jin et al., 2017; Massadeh et al., 2021; Sifrim et al., 2016). Five of these variants were reported as pathogenic or likely pathogenic; the remainder were considered as variants of uncertain significance (VUS) (Table S1; classification of variants generally derived from bioinformatic analysis and clinical information). Variants described as benign are excluded from this group. 84% (26/31) of the reported patients displayed CHD (Figure S1).

*PRKD1* (Protein kinase D1, also known as PKCμ) encodes a serine/threonine protein kinase, which is involved in a variety of fundamental cellular functions including cell survival, migration, differentiation, proliferation and adhesion (Barrio‐Hernandez et al., 2020; Bollag et al., 2018; Eiseler et al., 2009; Spasojevic et al., 2018; Sroka et al., 2016), Golgi transport (Baron & Malhotra, 2002; Maeda et al., 2001) and the regulation of RAS signalling (Su et al., 2018). Mutations in genes associated with RAS signalling are associated with CHD in humans and mice (Araki et al., 2004; Delogu et al., 2022). Consistent with this, according to *Bgee* multispecies' expression database (version 15.0; accessed 26 October 2023; https://www.bgee.org/search/genes) (Bastian et al., 2021), *Prkd1* in mouse is highly expressed in the cardiovascular system, including the adult atria, ventricles, aorta and valves. It is also expressed in the embryonic endocardial cushions. Although less is known about expression patterns in the human, it is known to be expressed in regions such as the adult left ventricle and aorta. Within the heart, PRKD has been found to be involved in mitochondrial morphology and function in cardiomyocytes, and apoptotic signalling (Jhun et al., 2018). PRKD is also associated with the phosphorylation of the sarcomeric protein cardiac troponin I (Cuello et al., 2007; Haworth et al., 2004; Martin‐Garrido et al., 2018), indicating that it is a regulator of cardiomyocyte contraction. Further, PRKD1 is known to regulate pathologic cardiac hypertrophy through its action on class II histone deacetylases (HDACs) and signalling via the *myocyte enhancer factor‐2* family (MEF2) and TBX5 (Fielitz et al., 2008; Ghosh et al., 2009, 2019; Kim et al., 2008; Vega et al., 2004), and plays a role in the subsequent K^+^ channel remodelling with the potential to cause arrhythmia (Bossuyt et al., 2022). It also plays a role in VEGFA‐induced angiogenesis (Di Blasio et al., 2010; Wang et al., 2008). Both PRKD1 and PRKD3 have been associated with the failing heart and are of pharmacological interest for clinical trials (Rasooly et al., 2023).

Taken together, these studies demonstrate a likely critical though uncharacterized role for PRKD1 in the embryonic heart. In order to obtain a greater appreciation for the role that this essential protein kinase plays in cardiogenesis and CHD, we have analysed a *Prkd1* transgenic mouse model (*Prkd1* ^*em1*^) carrying deletion of exon 2 (*em1*) which results in a premature stop codon prior to the critical functional domains, causing loss of function. Significantly reduced levels of *Prkd1* mRNA and protein were seen, with no compensation by the related *Prkd2* and *Prkd3*. We show that homozygous deletion of *Prkd1* is associated with severe forms of CHD and is lethal, either embryonically or in the early postnatal period, although survival to at least postnatal day 7 is possible. Morphological 3D analysis by high‐resolution episcopic microscopy (HREM) has shown a range of CHDs occur in the *Prkd1* ^*em1*^ mouse hearts at embryonic day (E)15.5 and the postnatal day (P) 6/7.

## 2 MATERIALS AND METHODS

### 2.1 Animal maintenance and genotyping

*Prkd1* ^*em1*(*IMPC*)*Wtsi*^ mice carrying a CRISPR/Cas9‐mediated deletion mutation of exon 2 (abbreviated to *Prkd1* ^*em1*^ in this study) were obtained from Wellcome Trust Sanger Institute. Wild‐type mice (C57BL/6N) were obtained from Charles River. Heterozygous mice for *Prkd1* ^*em1*^ (*Prkd1* ^*em1*/+^) were backcrossed with wild‐type (*Prkd1* ^+/+^) mice for colony maintenance, and with heterozygous mice for obtaining homozygous (*Prkd1* ^*em1*/*em1*^) embryos. The day of visualizing a vaginal plug was considered as E0.5. The day of birth was considered as P0.

Mice were genotyped by end point PCR. The cycling conditions comprised 5 min of polymerase activation at 94°C and 34 cycles of 94°C, 30 s; 58°C, 30 s; and 72°C, 1 min. Primer sequences are provided in Table S2. All animal procedures were performed in strict accordance with the Animals (Scientific Procedures) Act (ASPA), 1986.

### 2.2 Tissue collection

All animals were humanely culled. Embryos (E12.5 [*n* = 53] and E15.5 [*n* = 174]) were dissected and staged using a stereo microscope (Zeiss Discovery V8). For HREM, E15.5 (*n* = 117) and P6/7 (*n* = 21) hearts were dissected and washed in 37°C PBS, fixed in 4% PFA for 20 min, then washed in distilled water for less than an hour until clear of blood, and then fixed in 4% PFA at 4°C overnight. For RNA and protein studies, embryos were dissected in 4°C DEPC‐treated PBS, and tissues were snap frozen in liquid nitrogen and stored at −80°C.

### 2.3 RNA extraction and RT‐qPCR

Total RNA extraction was done using Qiagen RNeasy Micro kit (Cat#. 74,004) and treated with RNase‐free DNase I provided in the kit following the manufacturer's protocol. Extracted RNA was quantified by nanodrop spectrophotometer ND‐1000. A 260/280 of ~2:00 was considered pure. Extracted RNA was stored at −80°C or used directly for cDNA synthesis. For cDNA synthesis, 500 ng of RNA was reverse transcribed in 20 μL reaction using SuperScript™ II Reverse Transcriptase kit with random hexamers following the manufacturer's protocol (Cat. # 18064014), then diluted five times with nuclease‐free water, aliquoted, and stored at −20°C. A no‐reverse transcriptase reaction (RT‐) was included for each sample to assess for gDNA contamination. Quantitative real‐time PCR was done using SYBR green dye (iTaq Universal SYBR Green Supermix Bio‐Rad #1725121). In order to obtain a standard curve, samples were run in duplicates and six dilution points of 1:3 from stock cDNA to determine primer efficiencies, generate melt curves and determine the best dilution to use for qPCR. Efficiencies between 85% and 110% were accepted, with *R* ^2^ values >0.98. For relative quantification, three biological and three technical replicates, a no‐template control (NTC) and RT‐ controls were included in each plate. The reaction mix (10 μL) was manually set to contain 1 μL of stock cDNA, 250 nM of reverse and forward primers, and 5 μL of SYBR green. *Pgk1* and *Rpl4* were used as reference genes. Reactions were run on Applied Biosystems 7500 Fast Real‐time PCR system. The thermocycler conditions were similar for all reactions and comprised 50°C, 2 min; 95°C, 1 min; 40 cycles of 95°C, 15 s; and 60°C, 15 s; followed by melt curve stage that comprised 94°C, 15 s; 60°C, 1 min; 95°C, 30 s; and 60°C, 15 s. Primers for qPCR were designed and checked for specificity using primer3 and BLAST (Koressaar & Remm, 2007; Untergasser et al., 2012; Ye et al., 2012). Primers for reference genes were adapted as previously described (Ruiz‐Villalba et al., 2017) (see Table S2). PCR products were run on 2% agarose gel to check for the correct amplicon length.

### 2.4 Protein extraction and western blot analysis

For extracting protein from mouse heart, cold extraction buffer (Tris. HCl buffer‐pH 7.6 containing 8 M urea) was added to the tissue (1 mL/200 mg tissue) with 1× protease inhibitor (cell signalling technology, Cat. # 5872). The solution was homogenized for 20 s and centrifuged at 12,000*g* for 20 min at 4°C to collect the clear supernatant. After protein estimation by Bradford method (Biorad® Quick Start™ Bradford 1× Dye Reagent, Cat. # 5000205), 20 μg tissue extract was mixed with 1× LDS sample buffer (NuPAGE™, Cat. # NP0007) and 1× reducing agent (NuPAGE™, Cat. # NP0004). The solution was heated at 95°C for 5 min before loading onto the NuPAGE™ 10%, Bis‐Tris Protein Gel (Cat. # NP0301BOX, Invitrogen™). Proteins were separated using 1× NuPAGE MOPS SDS Running Buffer (Cat. # NP0001, Invitrogen™) at 200 V for 55 min. Precision Plus dual‐stain protein standard (Cat. # 1610376, Biorad®) was loaded to a well on one end of the gel. Proteins were then transferred from gel to a polyvinylidene fluoride (PVDF) membrane (Cat. #. LC2002, Invitrogen™) at 25 V for 90 min. For this purpose, 1× Tris‐Glycine transfer buffer (Cat. # LC3675, Invitrogen™) and Blot Module (XCell II™ Cat. # EI9051, Invitrogen™) were used. The membrane was then processed for immunodetection of the proteins. The primary antibodies used were the rabbit monoclonal anti‐PKCmu/PKD (ab51246, Abcam®) at 1:1000 dilution and rabbit anti‐GAPDH polyclonal antibody (ab9485, Abcam®) at 1:2500 dilution, both in 5% BSA‐TBST. Goat anti‐rabbit horse‐radish peroxidase (ab6721, Abcam®) was used as secondary antibody in 1:2000 dilution. For protein detection, Clarity Western ECL Substrate (Bio‐Rad, Cat. #1705061) was used, whereas the chemiluminescent images were captured digitally on a LAS‐3000 Imager (Fujifilm®). For relative quantification, three biological replicates per genotype and isoform were performed, with each having three technical replicates.

### 2.5 Embedding, sectioning and imaging

A detailed protocol is provided in supplementary information for the HREM. Hearts previously fixed in 4% PFA were washed in PBS, and then dehydrated with increasing concentrations of methanol (from 10% to 100%). Dehydrated hearts were embedded in JB4 methacrylate resin containing eosin and acridine orange (Sigma Aldrich EM0100‐1KT) as described previously (Weninger et al., 2018). Briefly, the samples were immersed in a 50:50 mix of 100% methanol:JB4 dye mix overnight, and then in pure JB4 dye mix overnight. The hearts were then placed in embedding blocks containing polymerizing JB4 dye mix, and left overnight to solidify at room temperature, and stored at 4°C. Before sectioning, hearts were baked at 95°C for 24 h and then kept at 4°C for 24 h. E15.5 hearts were sectioned at 2 μm thickness, and P6/7 hearts were sectioned at 3 μm thickness using high‐resolution episcopic microscopy (HREM) (Indigo Scientific). Images of block surface were captured by Jenoptik ProGres GRYPHAX microscope camera. The *z* stack of images obtained after sectioning was downsized, cropped and inverted using GraphicConverter 9, ImageJ and Adobe Photoshop, and were then visualized in three dimensions using OsiriX MD.

### 2.6 Data analysis

HREM‐sectioned hearts were initially evaluated blind to genotype by two researchers, followed by group analysis with four researchers for detailed morphological assessment. Reference genes for qPCR were validated using Refinder (http://blooge.cn/RefFinder/). qPCR standard curves and melt curves were examined using 7500 software v2.0.6. Comparative qPCR data were analysed using a modified Pfaffl method to normalize to two reference genes as described previously (Hellemans et al., 2007; Vandesompele et al., 2002). In case of negative control amplification, a difference of 10 Cq values or more between the RT‐ or NTC and the corresponding RT+ sample was accepted.

For quantitative data, one‐way ANOVA (*p* ≤ 0.05) was applied using GraphPad Prism 9.2.0 to compare the three groups followed by post‐hoc Tukey (multiple comparisons) test. In case of data not normally distributed, Kruskal–Wallis test was applied instead of one‐way ANOVA, followed by Dunn's multiple‐comparison test. In order to carry out lethality assessment, Chi‐square test was applied using MS‐Excel.

## 3 RESULTS

### 3.1 *Prkd1* was reduced in the *Prkd1^em1^* embryonic heart, but *Prkd2* and *Prkd3* expression were not up‐regulated to compensate

**FIGURE 1:**
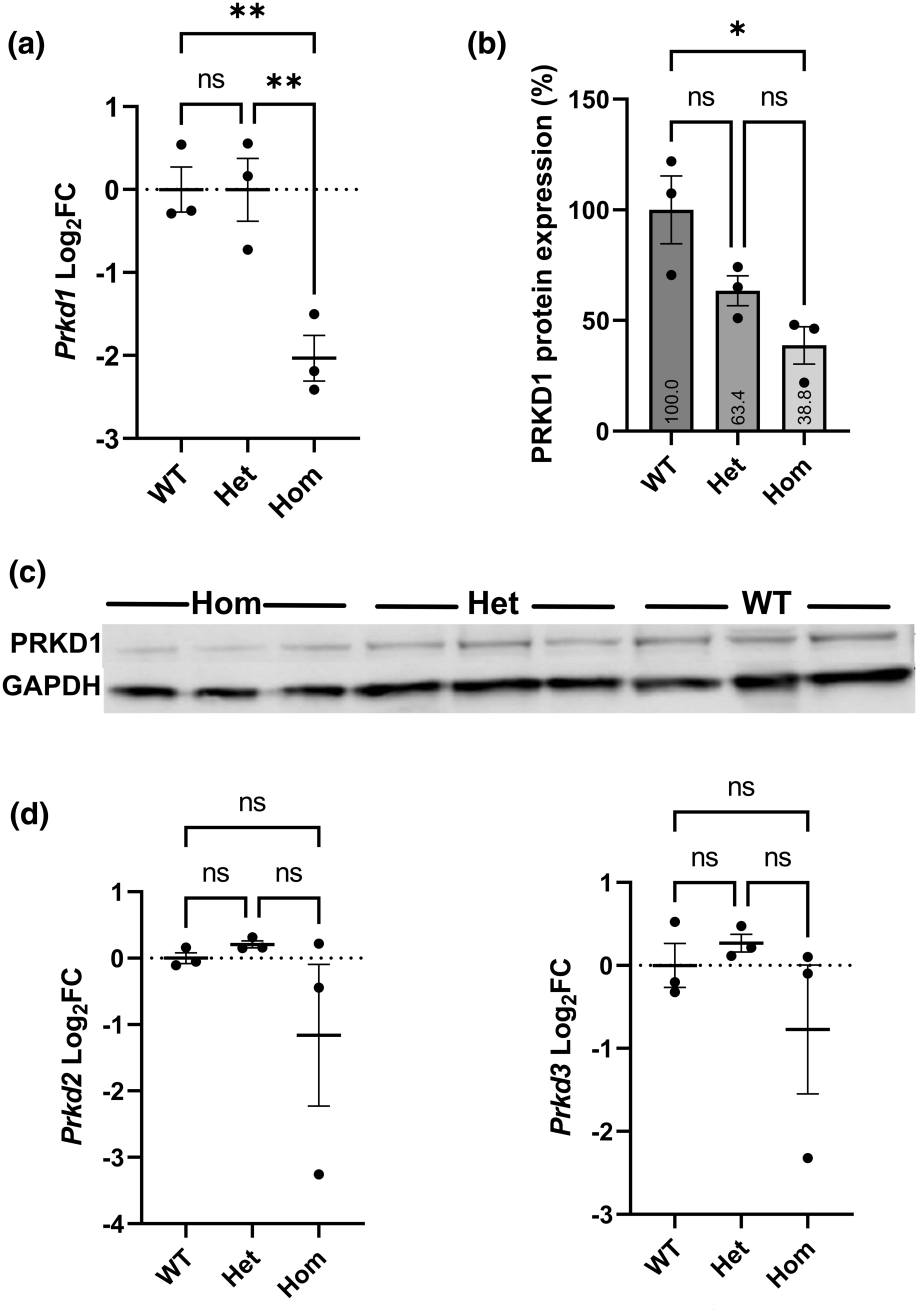
Expression of *Prkd1*, *Prkd2* and *Prkd3* in E12.5 hearts. (a) *Prkd1* mRNA expression in *Prkd1* ^*em1*/+^ (het) and *Prkd1* ^*em1*/*em1*^ (hom) hearts, compared to WT. A statistically significant difference in expression of *Prkd1* in homozygous hearts compared to wild type (WT) (*p* = 0.009) and heterozygotes (*p* = 0.009) was found. (b) Mean normalized PRKD1 protein expression in percentage. *Prkd1* ^*em1*/+^ (het) and *Prkd1* ^*em1*/*em1*^ (hom) hearts were compared to WT (100%) using GAPDH as the reference protein. A significant difference was seen between homozygotes and WT controls (*p* = 0.017). (c) Representative western immunoblot of PRKD1 and GAPDH loading control in *Prkd1* ^*em1*^ E12.5 hearts. (d) *Prkd2* and *Prkd3* mRNA expressions in *Prkd1* ^*em1*/+^ (het) and *Prkd1* ^*em1*/*em1*^ (hom) hearts, compared to WT. No statistically significant difference in expression of *Prkd2* or *Prkd3* in homozygous hearts compared to WT (*p* = 0.43 and 0.52, respectively) and heterozygotes (*p* = 0.33 and 0.34, respectively) was found. For all experiments *n* = 3 per group, with each study repeated three times; error bars denote SEM; FC, fold change; n.s, not significant; **p < 0.01; *p < 0.1.

In order to determine the level of *Prkd1* RNA expression in the *Prkd1* ^*em1*^ embryonic mouse heart, RT‐qPCR was performed on *Prkd1* ^*em1*/*em1*^ (homozygous), *Prkd1* ^*em1*/+^ (heterozygous) and *Prkd1* ^+/+^ (wild‐type [WT]) E12.5 hearts. There was a significant decrease in mRNA expression in the homozygous hearts compared to both heterozygous and WT controls (*p* = 0.009). However, there was no significant change in expression between heterozygotes and controls (*p* > 0.999) (Figure 1a; *n* = 3). Levels of PRKD1 protein were also determined in the E12.5 hearts, with three biological replicates analysed. A significant decrease (61.2%; *p* = 0.017) in PRKD1 protein was seen in homozygotes compared to WT controls (Figure 1b,c). In addition, intermediate levels of PRKD1 protein were seen in the heterozygotes although the difference from either homozygotes or wild type did not achieve statistical significance (Figure 1b,c). Furthermore, qPCR analysis of *Prkd2* and *Prkd3* did not show any significant difference in expression (Figure 1d; *n* = 3) in heterozygous (*Prkd1* ^*em1*/+^) E12.5 hearts. However, compared to the wild type and heterozygotes, increased variation between samples was detectable in mRNA expression in the homozygous hearts (Figure 1d), which was reproducible. This therefore suggests an impact of the PRKD1 mutant protein or its lower abundance on the regulation of expression of the other two isoforms.

### 3.2 A range of congenital heart defects were seen in *Prkd1* ^*em1*/*em1*^ homozygotes, with a smaller number in *Prkd1* ^*em1*/+^ heterozygotes

At E12.5, there was no significant difference between observed and expected numbers of homozygous, heterozygous and WT embryos (Table 1). There were significantly less homozygous *Prkd1* embryos at E15.5, compared to heterozygotes and WT (Table 1). Just 4.6% of embryos were homozygous (25% expected), compared to 62.07% heterozygous (expected 50%) and 33.33% of WT (25% expected) (*p* = 0. 0000000036). In addition to this, seven (out of a total of 60; 11.67%) reabsorbed embryos were found at E12.5 and 42 (out of 216; 19.44%) at E15.5.

**TABLE 1:** Ratios of E15.5 embryos from a *Prkd1* ^*em1*^ heterozygous x heterozygous cross.

Genotype	*Prkd1* ^*em1*^ (*Prkd1* ^*em1*/+^ X Prkd1^em1/+^)			
	E12.5		E15.5	
	Expected (%)	Observed (%)	Expected (%)	Observed (%)
WT	13.25 (25%)	12 (22.64%)	42.5 (25%)	58 (33.33%)
HET	26.5 (50%)	29 (54.72%)	85 (50%)	108 (62.07%)
HOM	13.25 (25%)	12 (22.64%)	42.5 (25%)	8 (4.6%)
	Chi‐square test	*p* = 0.79	Chi‐square test	*p* = 0.0000000036

*Note*: In addition to this, seven reabsorbed embryos were found at E12.5 and 42 at E15.5.

Abbreviations: Het, heterozygous; hom, homozygous; WT, wild type.

External analysis was performed on E12.5 (*n* = 53) and E15.5 (*n* = 174) embryos upon harvesting (before genotyping). External abnormalities were seen in 12 out of 20 (60%; 10 out of 12 E12.5 and two out of eight E15.5) homozygous and 4 out of 137 (2.9%; one out of 29 E12.5 and three out of 108 E15.5) heterozygous embryos. The abnormalities seen were growth retardation in 11 out of the 20 (55%) homozygotes and in 1 out of 137 heterozygotes. Further, oedema of the back was seen in one homozygote and two heterozygotes. Defects were not observed in WT embryos (*n* = 70; 12 E12.5 and 58 E15.5). With regards adult mice, ectodermal dysplasia was not assessed.

**FIGURE 2:**
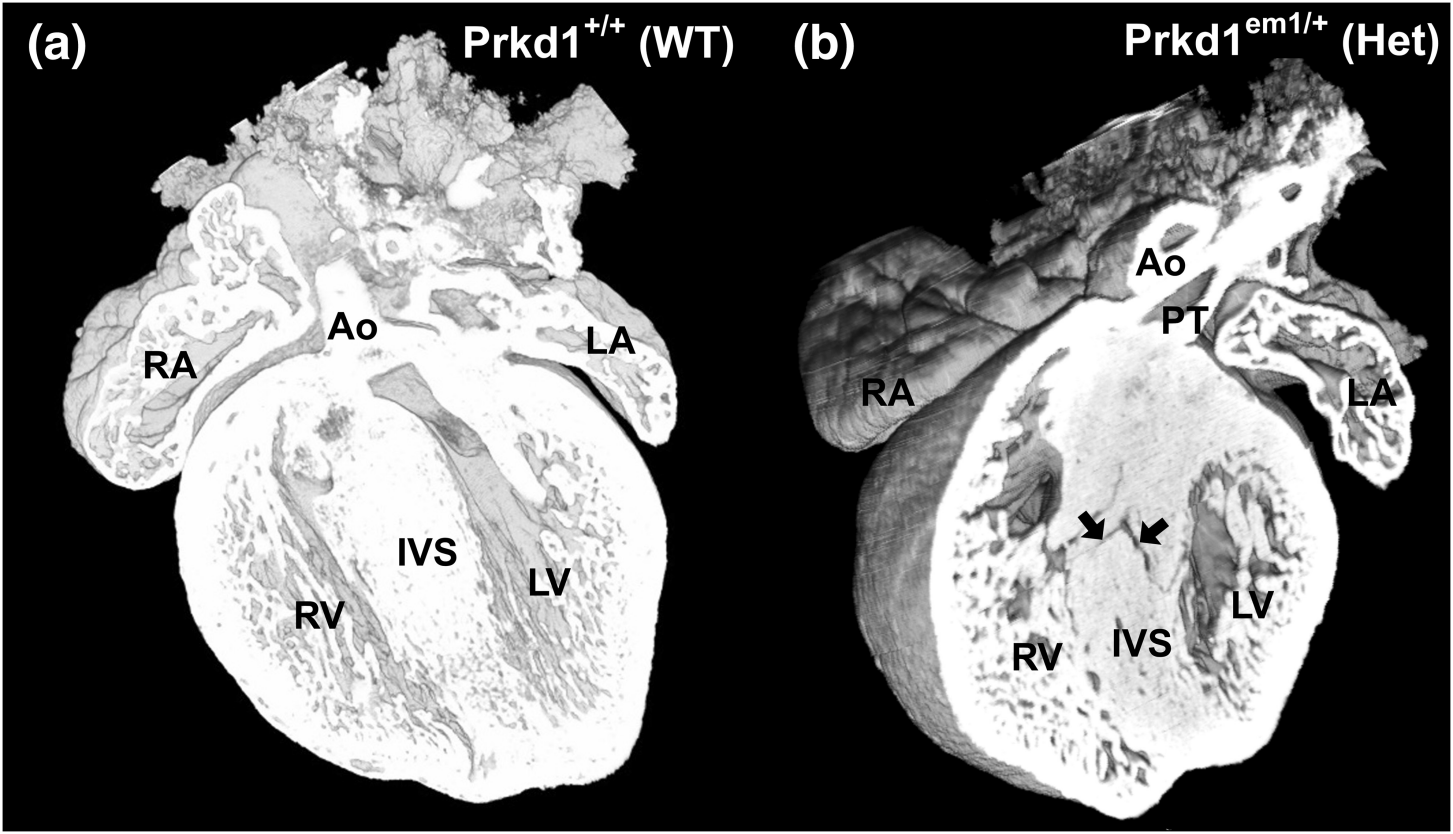
One heterozygous *Prkd1* ^*em1*^ E15.5 heart had a congenital heart defect. (a–b) Ventral view of a WT (a) and *Prkd1* ^*em1*/^ (b) heart. A small muscular ventricular septal defect can be seen in the *Prkd1* ^*em1*/+^ heart (arrows), in comparison to WT control where a normal interventricular septum (IVS) can be seen. The myocardial trabeculae also appear coarse and hypertrophic compared to controls. Ao, Aorta; LA, left atrium; LV, left ventricle; PT, pulmonary trunk; RA, right atrium; RV, right ventricle; WT, wild type.

To assess the incidence of CHD, HREM was performed at E15.5. At this stage, the outflow tract and the ventricular chambers have normally completed septation to form a four‐chambered structure with correctly aligned outflow vessels (Geyer et al., 2017), so most common CHDs should be detectable. A total of 56 WT, 55 heterozygous and six homozygous embryos were analysed by HREM (Table 2). Of the 55 heterozygous hearts at E15.5, just one had a CHD (1.82%). Internal analysis showed that there was a small muscular ventricular septal defect (VSD; an opening between the right and left ventricular chambers) (arrows in Figure 2b), a defect that was not seen in any of the 56 WT hearts (Figure 2a). In addition, the myocardial trabeculae appeared coarser and hypertrophic compared to control in this heart.

**TABLE 2:** Phenotypes in E15.5 *Prkd1* ^*em1*^ mouse hearts.

Genotype	Total	N with CHD (%)	Phenotype
WT	56	0	‐
HET	55	1 (1.82%)	Musc VSD
HOM	6	5 (83.3%)	Dysplastic PV (dysplastic left leaflet) BAV (fused type, with three sinuses) AVSD (with continuous inlet/outlet VSD), DORV AVSD (with continuous inlet/outlet VSD), DORV pMem VSD (outlet)

*Note*: Each row indicates a separate embryo.

Abbreviations: AVSD, atrioventricular septal defect; BAV, bicuspid aortic valve; DORV, double‐outlet right ventricle; het, heterozygous; hom, homozygous; musc VSD, muscular ventricular septal defect; pMem VSD, perimembranous ventricular septal defect; PV, pulmonary valve; WT, wild type.

**FIGURE 3:**
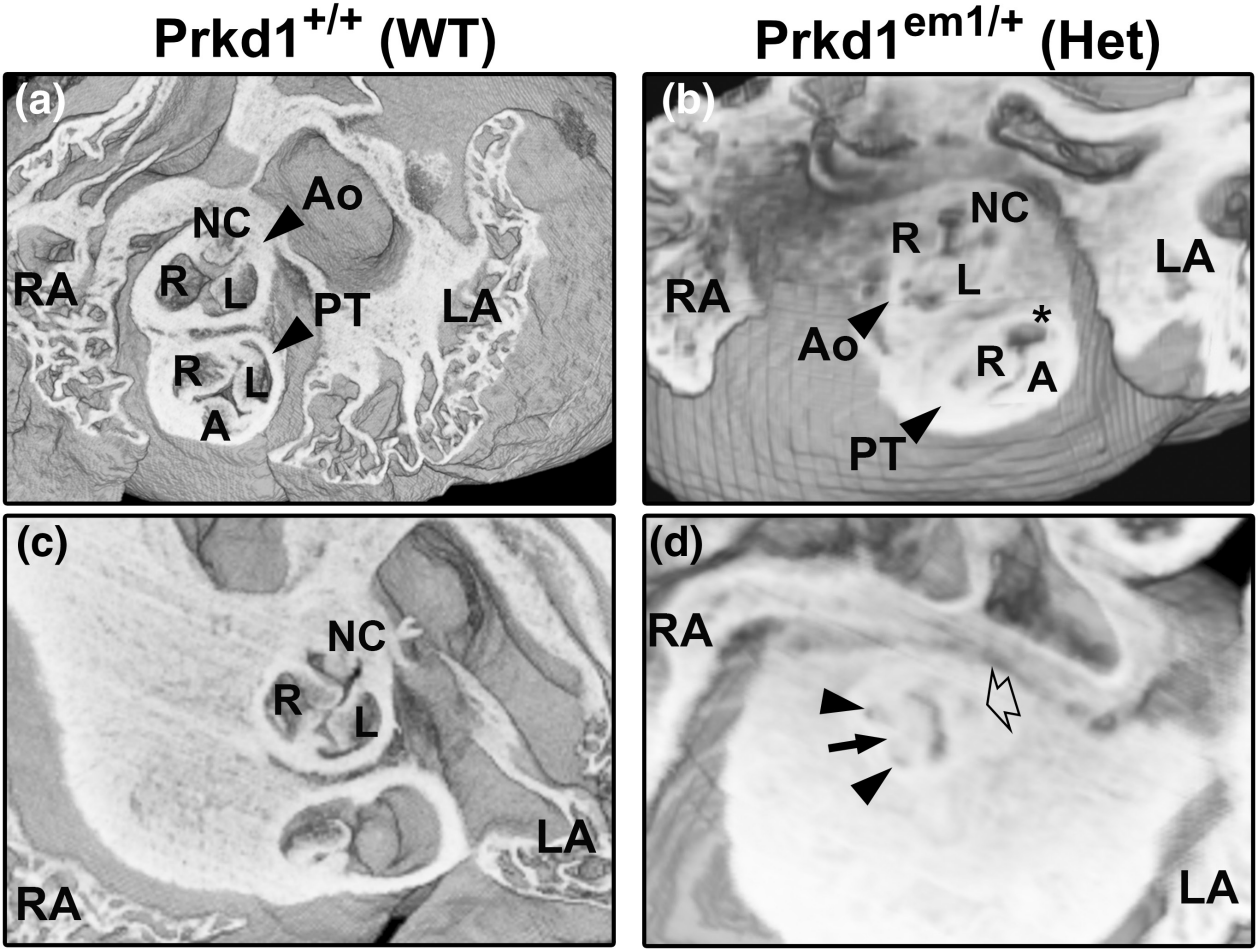
Morphology of abnormal valves in outflow vessels in E15.5 *Prkd1* ^*em1*/*em1*^ (homozygous) hearts. (a, b) Superior views of a *Prkd1* ^+/+^ (a) and *Prkd1* ^*em1*/*em1*^ (b) heart. The WT heart has normal valves in the outflow region, with three valve leaflets (right, left and anterior) seen in the pulmonary trunk (a). In contrast, the homozygous heart has an abnormal pulmonary valve (b); a right and anterior leaflet could be discerned, but the left leaflet was deficient (asterisk), indicating a dysplastic leaflet in the pulmonary valve. Three leaflets (right, left and non‐coronary) can be seen in the aortic valve in both hearts. (c, d) A second E15.5 WT heart (c) and homozygous (d) heart from the superior aspect. The WT heart has normal leaflets (right, left and non‐coronary) in the aortic valve (c). In contrast the homozygous heart had an abnormal aortic valve (d). There are three sinuses (trisinuate; two are denoted by small arrows and one by an open arrow), but there were only two leaflets (bileaflet). The long black arrow points to the site of raphe, where the right and non‐coronary valve leaflets are fused. A, anterior; Ao, aorta; L, left; LA, left atrium; LV, left ventricle; NC, non‐coronary; PT, pulmonary trunk; R, right; RA, right atrium; RV, right ventricle; WT, wild type.

In contrast to the heterozygotes, of the six E15.5 homozygous hearts, five had structural heart defects (83.3%, Table 2). The abnormalities seen in these five CHD‐affected hearts were variable, from isolated anomalies to more complex defects. One of the isolated defects was a perimembranous VSD seen in a single heart, and two additional hearts displayed abnormalities of the outflow valves; a dysplastic pulmonary valve and a bicuspid aortic valve (BAV) were seen in one heart each (Table 2). A normal pulmonary valve should have three leaflets: right, left and anterior leaflets (Figure 3a). In the heart with a dysplastic pulmonary valve, the anterior and right leaflets were present, but the left leaflet was deficient (Figure 3b). The aortic valve leaflets are normally arranged as right, left and non‐coronary, and these were readily seen in both the control and most of the homozygote hearts (Figure 3c). However, in one homozygote heart, the aortic valve was ‘bicuspid’, with fusion of the right and non‐coronary leaflets but with three separate sinuses (bileaflet and trisinuate) (Figure 3d). The long black arrow in Figure 3d indicates where the two leaflets had fused.

More complex defects were seen in the other two homozygous hearts (Table 2). An atrioventricular septal defect (AVSD) was noted in both. An AVSD forms due to a failure of fusion of one or more of the dorsal mesenchymal protrusion (vestibular spine), mesenchymal cap, atrioventricular (AV) endocardial cushions or primary atrial septum with the other components (Burns et al., 2016; Taqatqa & Vettukattil, 2021; Webb et al., 1998). This results in variations of a common AV junction and valve (Anderson et al., 2010). The septal deficiency and AV valve morphology in AVSDs is highly variable, in both the size of defects and the degree of separation of the common AV valve leaflets and their proportions (Franklin et al., 2017).

**FIGURE 4:**
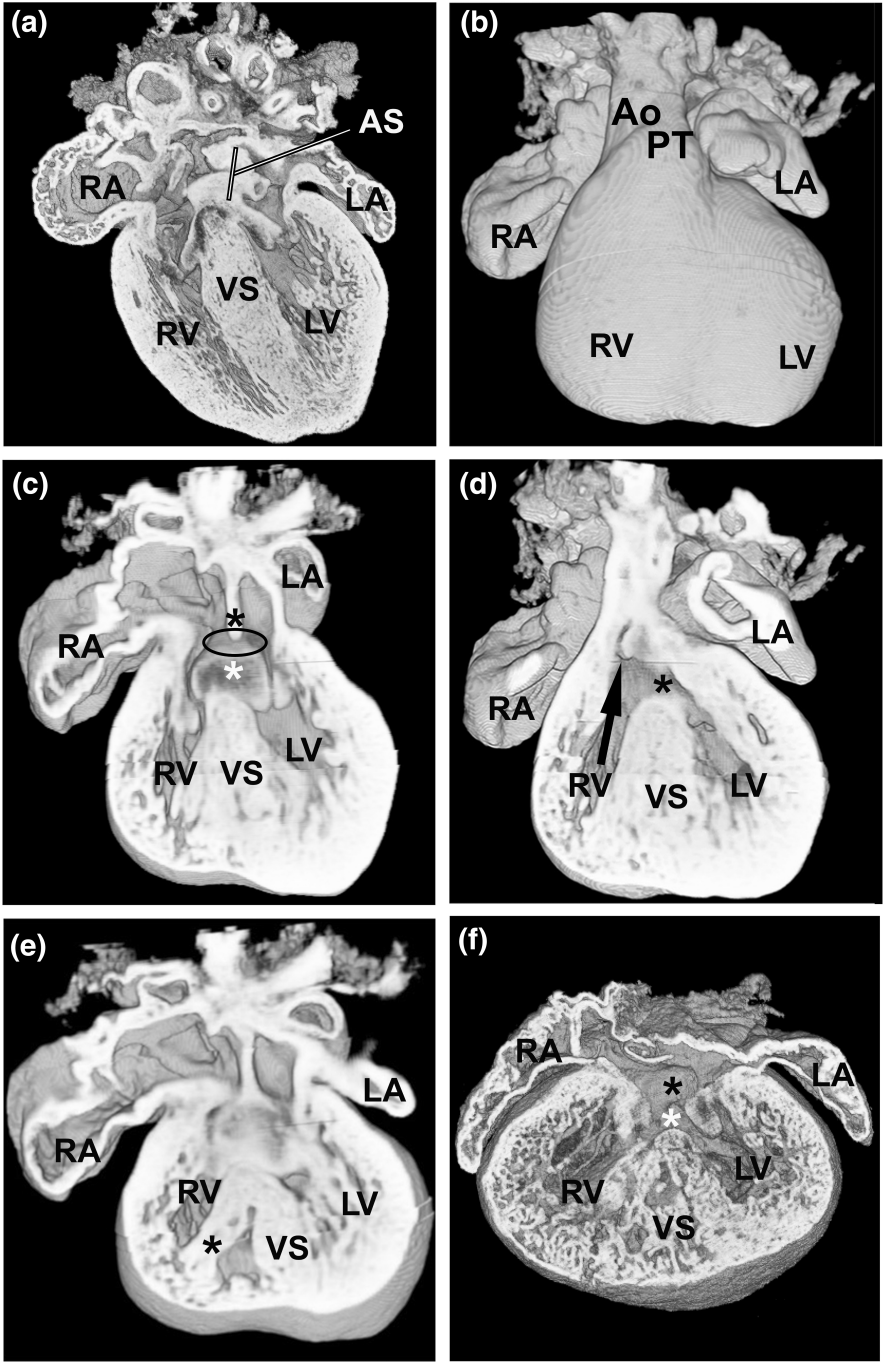
AVSD is seen in two E15.5 *Prkd1* ^*em1*/*em1*^ (homozygous) hearts. (a) A WT control heart with normal atrial septum. (b) An external ventral view of a *Prkd1* ^*em1*/*em1*^ heart with AVSD. (c) Same heart as in b; the lack of fusion of the dorsal endocardial cushion (white asterisk) with the septum primum (lack of fusion denoted by black oval) can be seen; the septum primum is indicated (black asterisk). The ventricular opening in this region is due to an inlet VSD. (d) A more ventral view shows the outlet VSD (black asterisk), with the aortic valve arising over the right ventricle (RV) (black arrow). The inlet and outlet VSDs are continuous. (e) A dorsal view, the right ventricle appears to be divided by either a large papillary muscle or a prominent septomarginal trabeculation (black asterisk). (f) Second homozygous heart with AVSD (black asterisk for atrial part and white asterisk for the inlet VSD). There is very marked trabeculation of the myocardium of both ventricles, with deep intertrabecular crypts and only a thin layer of compact myocardium present. There are a number of muscular openings within the ventricular septum. AS, atrial septum; Ao, aorta; LA, left atrium; LV, left ventricle; PT, pulmonary trunk; RA, right atrium; RV, right ventricle; IVS, interventricular septum; WT, wild type.

Figure 4 shows the homozygous E15.5 hearts with AVSD. In one of these hearts (Figure 4b), the septum primum was present (black asterisk in Figure 4c), but deficient (small) compared to wild type with an atrial septal communication present (denoted by black oval), compared to WT (Figure 4a). The dorsal endocardial cushion could be seen (white asterisk in Figure 4c), and there was an associated ventricular septal deficiency extending from the inlet septum to the outlet region of the heart (Figure 4d). In addition, the aortic valve overrides the crest of the ventricular septum of this heart by more than 50%, with the pulmonary trunk arising normally from the RV, but more anteriorly. The degree of aortic override (confirmed in other sections) is such as to be described as double‐outlet right ventricle (DORV). Furthermore, at the dorsal aspect the right ventricle appeared to be significantly subdivided by either a large papillary muscle or prominence of the septomarginal trabeculation (asterisk in Figure 4e), although there seemed to be more generalized thickening of all the trabeculae in this heart. In the second homozygous heart with an AVSD (Figure 4f), the septum primum could not be discerned (black asterisk in Figure 4f), also with a large deficiency of the inlet septum on the ventricular side (white asterisk in Figure 4f). This too extended into the outlet of the heart with DORV. The ventricular myocardium of both ventricles and the interventricular septum had coarse, deeply crypted trabeculae, with a number of openings present in the muscular septum (Figure 4f), in comparison to the normally septated WT heart (Figure 4a). The trabeculation was especially marked in this heart, with only a thin layer of compact myocardium present. To date, this has not been noted in patients, but clearly these are all in individuals who are significantly ‘older’ than the mice in our study.

### 3.3 Defects were seen in postnatal hearts

**FIGURE 5:**
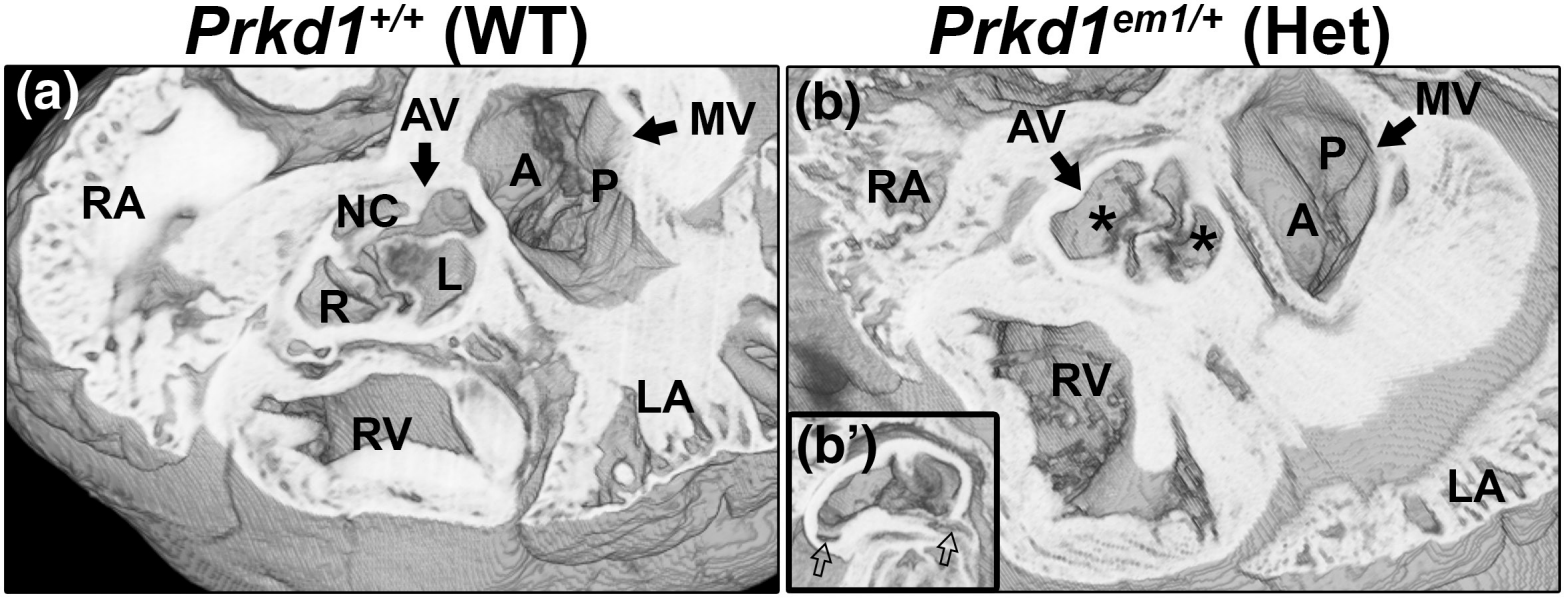
Bicuspid aortic valve is seen in P7 *Prkd1* ^*em1*/+^ heart. (a) Axial view of a *Prkd1* ^+/+^ (WT) heart at P7 stage showing a normal aortic valve with three sinuses of the three leaflets. (b) A *Prkd1* ^*em1*/+^ (Het) heart with a bicuspid aortic valve (AV), with two sinuses from the two leaflets (right and left) denoted by an asterisk (bisinuate and bileaflet). The mitral valve (MV) is also denoted, with normal appearing anterior (A) and posterior (P) leaflets. (b′) Inset in b is taken at a slightly different angle to show the right and left coronary ostia are patent (open arrows). R, right; L, left; NC, non‐coronary; Ao, aorta; LA, left atrium; LV, left ventricle; RA, right atrium; RV, right ventricle; WT, wild type.

Postnatal hearts were isolated in order to determine the phenotypes present after birth. Ten P6/P7 heterozygous hearts were analysed by HREM, with one showing a CHD (Table 3). This heart clearly showed a BAV with two sinuses and leaflets (bisinuate and bileaflet; right and left leaflets), best visualized from a superior view of the heart (asterisks in Figure 5b) in comparison to the WT control where the normal three leaflets (right, left and non‐coronary) can be seen (Figure 5a). The origins of the right and left coronary arteries from the two sinuses can be seen to be patent (open arrows in the inserted b′ in Figure 5b). Both of the coronary arteries could be followed till they curved, to the posterior aspect of the heart for the right coronary artery, and until the left coronary artery branched into the circumflex and anterior interventricular arteries. As described above, BAV is a developmental defect, and we did see it also in an E15.5 homozygous heart (Table 2; Figure 3d).

**TABLE 3:** Phenotypes in P6/P7 neonatal *Prkd1* ^*em1*^ mouse hearts.

Genotype	Total	N with CHD (%)	Phenotype
WT	10	0	‐
HET	10	1 (10%)	BAV (two‐sinus type)
HOM	1	1	Abnormal RV axis with widely spread bilateral apices, small RV and LV cavities, thickened myocardium, likely biventricular outflow tract narrowing.

Abbreviations: BAV, bicuspid aortic valve; het, heterozygous; hom, homozygous; LV, left ventricle; RV, right ventricle; WT, wild type.

**FIGURE 6:**
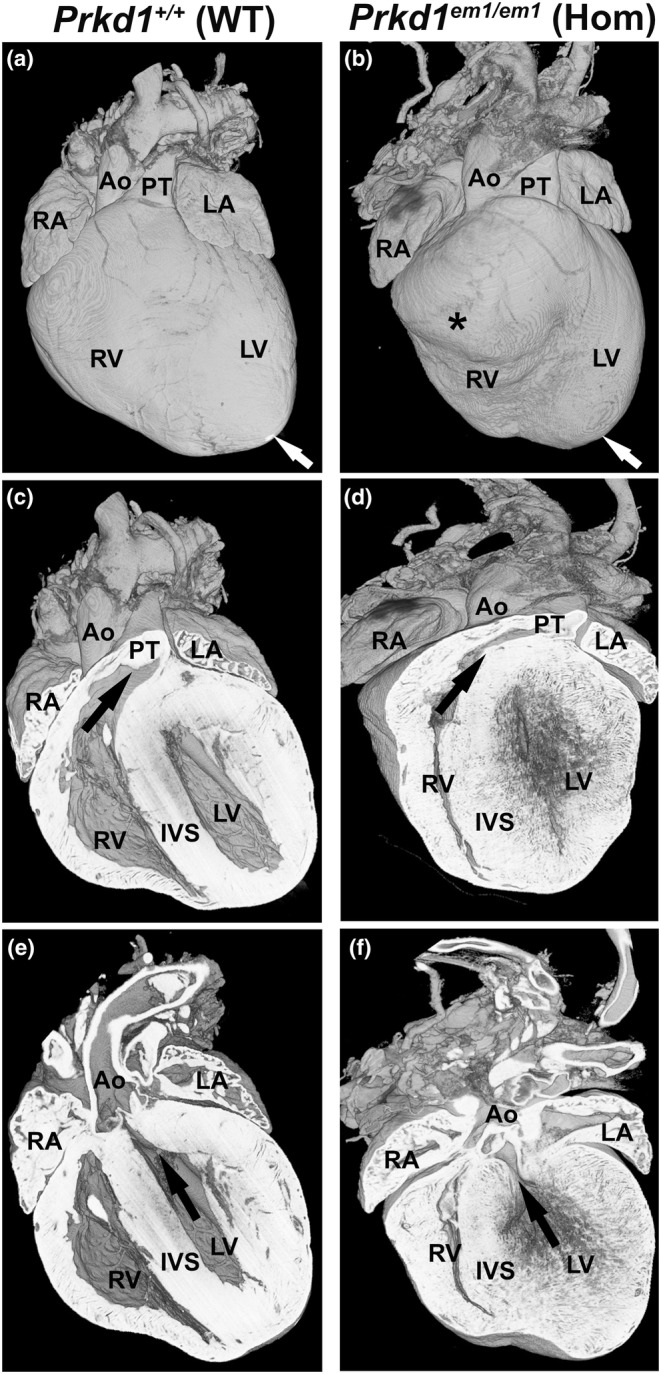
Comparison of *Prkd1* ^+/+^ and *Prkd1* ^*em1*/*em*1^ hearts at P7. (a, b) External view of *Prkd1* ^*em1*/*em1*^ (homozygous) heart shows that it is smaller and appears to have a more rounded shape and bifid apices (white arrow and asterisk), with the right ventricular apex located ventrally and further to the right in comparison to *Prkd1* ^+/+^ (WT) control. (c, d) Ventral view showing a reduced cavity size in the RV body as well as the RV outflow tract, which is longer and more horizontally orientated than in the WT heart (black arrow). (e, f) Coronal sections (ventral view) of the same WT and the homozygous hearts oriented in a plane to show the LV cavity is small with poorly defined endocardial surface and marked myocardial thickening. Even allowing for the heart being ‘empty’ there is an increase in subaortic crowding than in the WT heart, consistent with a left as well as right ventricular outflow tract narrowing. Ao, aorta; LA, left atrium; LV, left ventricle; PT, pulmonary trunk; RA, right atrium; RV, right ventricle; IVS, interventricular septum; WT, wild type.

Although we have found that the *Prkd1* ^*em1*^ homozygous embryo can survive to E15.5, they do so at reduced frequency compared to controls, with just 4.7% of the collected embryos being homozygous (Table 1). We have never had a homozygous mouse surviving to notching, that is, when an ear notch is taken for genotyping at around P10–12. However, in this postnatal study, one of the P7 hearts was homozygous (Table 3). On external examination, this heart had a more rounded appearance with prominent and separate right and left ventricular apices (Figure 6b and Figure S4b) in comparison to WT (Figure 6a; Figure S4a). Although dual apices are seen in the normal developing mouse heart (Spicer et al., 2022), the position of the right ventricular apex in this heart was abnormal. This heart was processed with other hearts which were found to be morphologically normal, and there was no suggestion that this heart had acquired a separate processing artefact; this heart was not mechanically damaged. Therefore, we believe that the abnormal morphology of this P7 heart was due to its genotype. The course of the anterior interventricular coronary artery was abnormal in this homozygous heart (black arrows in Figure S4b, compared to control Figure S4a). It is seen to originate from the right coronary artery rather than, as is more usual, from the left coronary artery; it then traverses across the right ventricular outflow tract and runs between the right and left ventricular chambers in the anterior interventricular groove. Additionally, the right and left ventricular cavity sizes appear small with marked thickening and poorly defined trabeculation of the myocardium of both ventricles compared to control (Figures 6d,f compared to c,e). The axis of the right ventricle was much more vertical than usual, consistent with the position of the right ventricle apex. Although the left and right ventricular cavities connected anatomically correctly with the aorta and pulmonary trunk, respectively (Figure 6d,f in comparison to c,e), there is much more subaortic (and subpulmonary) crowding than usual consistent with biventricular outflow tract narrowing. Septal defects were not apparent (Figure S4d compared to control Figure S4c). Having only one homozygous postnatal heart available for analysis limits interpretation, but phenotypically this heart could be viewed as having some features resembling the bilateral outlet obstruction and cardiomyopathy seen in severe (and often lethal) Noonan's syndrome or other RASopathies (Lioncino et al., 2022; Marino et al., 1995).

## 4 DISCUSSION

CHD is highly prevalent and accounts for a third of all major congenital defects. Due to improvements in surgical and medical care, the number of adults with CHD has rapidly increased, often needing lifelong medical care. BAV is the most common CHD, occurring in at least 2% of the population, with a 2:1 male‐to‐female preponderance. Although it may never cause symptoms, in more severely affected individuals, it is associated with progressive aortic valve stenosis and/or regurgitation, aortic coarctation and a more generic ‘aortopathy’ for which the understanding of the genesis is still evolving (Lioncino et al., 2022; Shah et al., 2018). Such individuals can require repeated surgeries or develop heart failure, aortic dissection and sudden death. There is an inherited component to the occurrence of BAV (Bravo‐Jaimes & Prakash, 2020). This is consistent with our findings; of the 72 *Prkd1* ^*em1*^ heterozygous and homozygous hearts analysed, two had a BAV. There are a number of different systems used to describe the anatomical variations in aortic valve morphology. The Sievers classification has been used primarily for surgical repair and is dependent on the number of raphes (Sievers & Schmidtke, 2007). In addition, Michelena and colleagues have described the three main types of BAV, which vary in the morphology of the leaflets and number of sinuses (Michelena et al., 2021). Standardization of terms was provided by the International Paediatric and Congenital Cardiac Code and the Eleventh iteration of the International Classification of Diseases (Jacobs et al., 2021). Importantly, this has recently been further amended by Tretter and colleagues (Tretter et al., 2023a, 2023b). HREM is a valuable technology for morphological analysis. For example, for valves of the outflow region, it provides enough resolution to confirm whether the correct number of sinuses are present, and make an assessment of the number of raphes. This is important in allowing the correct classification of the valve defect.

It is intriguing that the two hearts in this study that had BAV had two different types of defect that are considered to form via different mechanisms. One P7 heterozygous heart had a two‐sinus type of BAV (bisinuate, bileaflet), with a patent left and right coronary arteries emerging correctly from the left and right aortic sinuses, respectively (Figure 5). The absent leaflet was the non‐coronary one. This type of BAV accounts for 5%–7% of cases in humans (Michelena et al., 2021). The non‐coronary leaflet is derived from the intercalated leaflet swellings (sometimes called intercalated valve cushions); it has been proposed that it is a lack of these structures that results in the two‐sinus type of BAV (Anderson et al., 2014). Absence (or dysplasia) of the non‐coronary leaflet has been associated with SOX17‐PDGRB signalling (Lu et al., 2022). The other heart with BAV was seen in an E15.5 homozygote; the aortic valve appeared to be of the fused type, with the right and non‐coronary leaflets fused (Figure 3d). This type of BAV has been seen in *Gata5* and *Nos3* null mutant mice (Laforest et al., 2011; Peterson et al., 2018). The fused types of BAV are the most common in humans, accounting for 90%–95% of cases, with fusion of the right and non‐coronary leaflets accounting for 20%–30% of these cases (Michelena et al., 2021). Fusion of the right and left coronary leaflets is more common, accounting for around 75% of fused BAV (Sillesen et al., 2021), a type that was not seen in any *Prkd1* mutants here. Abnormal *Gata6* expression in mice has been associated with this type of BAV (Gharibeh et al., 2018). Further, there are a number of other genes that have also been associated with BAV (Pasipoularides, 2019), including *Notch1* (Foffa et al., 2013). Seeing two different types of BAV with distinct molecular mechanisms is difficult to rationalize, with direct associations with PRKD1 not currently known. Little is known of downstream targets of PRKD1 in the heart and hence is an area that requires further study.

In addition, one homozygous E15.5 heart had a dysplastic pulmonary valve; this could result in pulmonary stenosis (PS) (or incompetence, or both), reported to occur in 8% of individuals with a CHD (Koretzky et al., 1969; Van Der Linde et al., 2011), and as described below is the most common defect in individuals with a heterozygous *PRKD1* mutation.

Aside from BAV, the next two most common CHDs in humans are ventricular septal defects (VSD) and atrial septal defects (ASD) (Liu et al., 2019). When isolated these are usually at the less severe end of the CHD spectrum, as even if haemodynamically significant they can usually be closed at low risk with a good long‐term prognosis. VSDs can occur in the perimembranous (depending on their location, described as inlet or outlet) or muscular regions of the ventricular septum. Two E15.5 embryos had an isolated VSD, one each of outlet perimembranous and mid‐muscular in a homozygous and a heterozygous *Prkd1* ^*em1*^ heart, respectively (Table 2). However, defects are occasionally seen in wild‐type embryos (Wilson et al., 2016); hence, a much larger sample size would be needed to assess whether seeing a VSD in a heterozygous heart is meaningful or simply a chance finding.

In two homozygous E15.5 hearts, an AVSD was seen. AVSD occurs in approximately 0.05% of live human births; approximately 50% of these are found in individuals with Trisomy 21 (Calkoen et al., 2016). Variants of VEGFA and genes in its pathway have previously been associated with the occurrence of AVSD in both syndromic and non‐syndromic individuals (Ackerman et al., 2012; Redig et al., 2014). PRKD1 has been shown to be a target of VEGFA by mediating VEGF‐induced endothelial cell proliferation and migration (Di Blasio et al., 2010; Wang et al., 2008). Both of the mouse hearts with AVSD had outlet extension of the VSD and overriding of the aorta to the extent of producing DORV, as a result of failure of the aortic root to transfer to the left ventricle sufficiently (Anderson et al., 2016). The great arteries are, however, normally related. DORV or an overriding aorta has been previously reported in mice with an AVSD (Anderson et al., 1998; Lin et al., 2020).

A phenotype seen in some hearts was hypertrophic trabeculae, which has previously been seen in other pathological conditions (Fatemifar et al., 2019; Loukas et al., 2013; Van De Veerdonk et al., 2014). In addition, in one E15.5 homozygous heart with a CHD in our study (see Figure 4f), there was marked trabeculation of the ventricular myocardium, with deep intertrabecular crypts and only a thin layer of compact myocardium present. A recent review has described a number of genes presenting with excessive trabeculation as part of the phenotype, including sarcomeric, mitochondrial and ion channel genes (Petersen et al., 2023). Conversely, excessive trabeculation can vary in its presentation and may not always be associated with a pathological condition (Petersen et al., 2023).

It has previously been described that homozygous deficiency of *Prkd1* in mice is embryonically lethal, though at reduced penetrance (Fielitz et al., 2008; Matthews et al., 2010). Our data support this, with only one homozygous mouse found by P7. We propose that residual amounts of protein (as shown in Figure 1b,c) are sufficient to permit some survival. Of the homozygous embryos at E15.5 and the mice sacrificed at P6/7, six (out of seven) had a CHD, of which several were severe. This contrasts with the heterozygotes, where 2 out of 65 had a CHD. However, the CHDs seen in this study by themselves would not result in embryonic lethality. The heart, which forms alongside the vascular system, is the first organ to develop in the embryo, and embryonic lethality is usually caused by mutations affecting the cardiovascular system at critical stages of development (Copp, 1995; Papaioannou & Behringer, 2012). The thin compact myocardium seen in the homozygous hearts at E15.5 could contribute to the lethality of homozygous embryos. A thin myocardium has been reported in the literature in association with a number of gene mutations (Khadhraoui et al., 2023; Rossant, 1996) including with Cdk13 (Nováková et al., 2019).

We also know that there was already embryonic attrition of homozygotes by E15.5. Therefore, analysis earlier in development could have been informative. We do not know about embryonic survival between E15.5 and birth or the early postnatal period, due either to the mothers not looking after pups that were failing to thrive or to pups that died upon closure of the ductus arteriosus. Interestingly, there was no significant decrease in *Prkd1* mRNA or protein levels in the *Prkd1* ^*em1*^ heterozygous mice, which could explain the low penetrance of structural heart defects in these embryos. The reduction of *Prkd1* expression in *Prkd1* ^*em1*^ homozygotes was observed at transcript and protein levels. There was no compensation by an up‐regulation in *Prkd2* and *Prkd3* expression, although the lack of compensation by these related genes is perhaps not surprising given that previous studies have shown that PRKD1 can act independently of PRKD2 and PRKD3 (Guo et al., 2011; Qiu & Steinberg, 2016). Nevertheless, there was a substantial variation in expressions of *Prkd2* and *Prkd3* in the *Prkd1* homozygous hearts; the low number of biological replicates performed (*n* = 3) is attributed to the low availability of homozygous tissue due to embryonic lethality. Of note, the analysed heart samples clustered in two distinct populations in both of these RT‐qPCR studies. Two homozygous hearts were similar to WT and heterozygous hearts. However, one heart did show a noticeable and reproducibly observable decrease in expression for both genes. This might indicate that *Prkd1* does indeed impact its own and/or related isoform expression, and the variable expression of these genes could be a factor in the penetrance and variation in phenotypes. This phenomenon has been previously reported as a regulatory feedback mechanism employed by protein kinase C beta II (Cejas et al., 2005). Furthermore, it has been reported that dimerization of two PRKD1 monomers, or between PRKD1 and the monomer of another PRKD, affects kinase activity and functions (Reinhardt et al., 2020), which warrants further investigation.

Of the 31 reported patients with different heterozygous *PRKD1* variants in humans that are documented in public repositories ClinVar (Landrum et al., 2018), Decipher (Firth et al., 2009) and in the literature (Alter et al., 2021; Jin et al., 2017; Massadeh et al., 2021; Sifrim et al., 2016), 26 (~84%) are reported to have a CHD phenotype. However, nine of these individuals were described as having CHD with ectodermal dysplasia, with details of the CHD not provided. Where the type of CHD is provided, coarctation and BAV were seen in one patient, AVSD was described for four and PS was present in five individuals. Another four individuals displayed more complex combinations of malformations.

Mutations in RAS/MAPK cell signalling pathway, or ‘RASopathies’, are associated with PS; these include Noonan and Leopard syndromes (Delogu et al., 2022; Tartaglia et al., 2011). PRKD1 is also associated with the regulation of RAS signalling (Su et al., 2018). Interestingly, RAS/MAPK regulates MEF2 resulting in hypertrophic cardiomyopathy (HCM) in some individuals with RASopathies (Yi et al., 2022). PRKD1 also regulates MEF2c (Fielitz et al., 2008; Kim et al., 2008). Further, RASopathies are associated with specific cancers (benign or malignant) (Rauen, 2022). PRKD1 has been shown to be downregulated in some types of cancers and overexpressed in others, and is associated with many cancer‐related signalling pathways (Sundram et al., 2011). Many of the CHDs and myocardial differences seen in Noonan syndrome and other RASopathies were seen in the *Prkd1* ^*em1*^ hearts, including AVSDs, VSDs and possible PS (Delogu et al., 2022, Tartaglia et al., 2011), in addition to bilateral outlet obstruction and cardiomyopathy (see Figure 6d,f), which can be seen in Noonan's syndrome and other RASopathies (Lioncino et al., 2022; Marino et al., 1995).

Therefore, taking together all these factors, we suggest that *Prkd1* may be another gene associated or involved with RASopathy cardiac phenotypes (Rauen, 2022). However, although we have seen a dysplastic pulmonary valve in one heart which could lead to PS, a limitation of this study has been our inability to determine the full postnatal penetrance of PS in the *Prkd1* ^*em1*^ hearts. PS usually occurs due to a reduced diameter, number of leaflets and/or opening of the pulmonary valve, although narrowing may also occur above or below the valve. In life, diagnosis is performed by an echocardiogram, which allows the structure of the pulmonary valve to be seen and the location and severity of the valve narrowing assessed in real time (Cuypers et al., 2013). Echocardiography was beyond the scope of this study, and so confirmation of functional PS was not performed. Further, PS does vary in severity and may only manifest in infancy or early childhood. Unfortunately, even critical PS can be difficult to detect prenatally during the second‐trimester screening (Ronai et al., 2020). In addition, optimal resolution was not achievable for all hearts, making a detailed analysis of some structures more difficult. Some of the hearts, in particular, the homozygous hearts with complex defects, had poorer resolution on HREM. This might have been due to some blood remaining within the ventricular chamber (possibly related to the increased trabeculation) despite numerous washing steps, which had effectively been cleared in other hearts processed at the same time.

PRKD1 plays a number of critical roles in the adult heart, such as in cardiac remodelling (Steinberg, 2021) and in heart failure (Rasooly et al., 2023). This study demonstrates that other *Prkd*‐related genes do not compensate for the deletion of *Prkd1*. Even in heterozygotes, cardiac differences can occur, albeit at low penetrance. However, in homozygous *Prkd1*‐deleted mice, more complex CHDs occur with high penetrance and myocardial abnormalities are also seen. Further research needs to be conducted to determine if *Prkd1* can be truly considered as a gene associated with RASopathies or simply bears some similarities.

## AUTHOR CONTRIBUTIONS

JDB and SL conceived the idea and designed the experiments, whereas QWU, AW and SR performed the experiments. QWU, FB, AW, AA, SR and SL performed data analysis. MPH, GD and FC provided additional intellectual input during the project. SL and AA wrote the first draft. All authors reviewed and edited the manuscript, and consented to the submission of the final draft.

## FUNDING INFORMATION

We would also like to express our appreciation to the Higher Education Department, KPK, Pakistan, who have funded QWU, the BHF for funding AW a British Heart Foundation Clinical Research Training Fellowship (FS/14/51/30879) and Hashemite University for funding AA.

## CONFLICT OF INTEREST STATEMENT

The authors declare they have no conflicts of interest.

## Supporting information


Data S1.

## Data Availability

Data available on request from the authors.
