# Supplementary material for: Effect of deletion of the protein kinase PRKD1 on development of the mouse embryonic heart
Source: J Anat. 2024 Feb 28;245(1):70–83. doi: 10.1111/joa.14033 (PMC11161829; doi:10.1111/joa.14033)
Supplement: Supplementary file 1 — Data S1. [file JOA-245-70-s001.pdf]

Supplementary information

Materials and methods

HREM embedding, sectioning, and imaging

Dehydration

Heart samples were placed in a labelled 24-well plate and washed with 4 repeated changes of PBS for 30 minutes per wash at room temperature. Samples were agitated on a shaker through the process. Hearts were then dehydrated using methanol series (10%, 20%, 30%, 40%, 50%) for 1 hour per step, and left at 50% step overnight in a plate sealed with cling film at 4°C. Dehydration was then continued with (60%, 70%, 80%, 90%, 95%, 100%) methanol, for 1 hour/step.

Infiltration

Samples were immersed overnight in 50:50 mix of methanol:JB-4 dye mix at 4°C. Samples were then rinsed and immersed in several mls of fresh JB-4 dye mix and left overnight at 4 °C on a shaker. Infiltration was done for 24 hours/step for E15.5 hearts, and for 48 hours/step for P6/P7 hearts.

Embedding

To embed the hearts in JB-4 dye, solution b was added to cold JB-4 dye mix, which allows it to polymerize. Special designed moulds were used to embed the hearts. A small amount of polymerization mix was added to the moulds first, and then the hearts were inserted in a known order, with the apex positioned towards the user, and as close to each other, as possible, but without touching (Figure S2A). For the E15.5 hearts, three hearts were embedded in one mould, and for P6/7, two hearts were embedded together. More polymerization mix was added on the top of the hearts and a labelled plastic chuck was placed on top showing the block ID and position of the hearts (Figure S2B). The top of the chuck was filled with polymerization mix, making sure the central part was also filled (Figure S2C). After an hour, a layer of oil was added on top of the blocks to allow them to solidify in oxygen free environment and left under a ventilated hood overnight. Blocks were then taken out of the mould and stored at 4 °C.

Sectioning and imaging

HREM used for sectioning is an O.H.R.E.M by Indigo Scientific (Figure S3). The camera to capture images is Jenoptik ProGres GRYPHAX microscope camera, and the computer software is Optical HREM 1.12.6.1. Before sectioning, the hearts were baked at 95 °C for 24 hours, then taken out to store at 4 °C for 24 hours. To section the hearts, the block is placed under an illuminated stereomicroscope and the depth of the block is measured using a ruler. A bounding box that contains the hearts is then scratched on the block surface to focus the image, and a scratch to mark the orientation of the block. The block is then placed in the microtome and position of the blade is adjusted to be just above the surface of the block. The position of the block is then adjusted to have the camera focused on the bounding box, and the resolution to be the best. Starting the sectioning, preparation sections are taken with a thickness of 10 µm, until the surface of the hearts is reached, then thickness is changed to 2 µm for E15.5 hearts, and 3 µm for P6/7 hearts (Figure S3), and depth is entered as measured in the beginning. A vacuum suction pump is used to collect the sections from the surface of the block to prevent it from obscuring the images. An image is taken to show the orientation of the block. Images that are taken now are saved on the computer and will be ready for further processing. At the end of the block a picture of the graticule is taken.

3D model production

To produce three dimensional models of the hearts, images from each block were downsized to 50% using imageJ. Then, using photoshop, an action was created for each heart in the block separately where it was cropped, underwent levels and curves adjustment to remove any black background, then inverted, and converted to 8 bit. The image used to set the action was closed without saving changes. Then using automate feature, all sections were edited using the action created for that specific heart and saved in a separate folder. When the edited sections were ready, they were imported into Osirix MD and visualized in 3-dimensions. Using the crop feature it is possible to scroll through the interior of the heart in different planes.

Table S1. Previously published patients harbouring *PRKD1* variants. Pathogenic, likely pathogenic and variants of uncertain significance were assembled from Clinvar, Decipher and the outlined publications. Cardiac phenotypes are underlined.

Sample/ Variant-ID	Source	Variant	Reported classification	Phenotype
**II-3**	Massadeh et al. PMID 33919081	NM_001330069.1: c.265-1G>T p.(?) (Homozygous)	Pathogenic	Septal defect
**III-1**	Massadeh et al. PMID 33919081	NM_001330069.1: c.265-1G>T p.(?) (Homozygous)	Pathogenic	Pulmonary stenosis
**III-2**	Massadeh et al. PMID 33919081	NM_001330069.1: c.265-1G>T p.(?) (Homozygous)	Pathogenic	TA type II and VSD, respiratory distress
**III-3**	Massadeh et al. PMID 33919081	NM_001330069.1: c.265-1G>T p.(?) (Homozygous)	Pathogenic	ASD
**LL-4**	Massadeh et al. PMID 33919081	NM_001330069.1: c.265-1G>T p.(?) (Homozygous)	Pathogenic	Septal defect
**1032083**	Clinvar	NM_002742.3:c.317A>G (p.His106Arg)	VUS	Congenital heart defects and ectodermal dysplasia
**272461**	Decipher	NM_002742.3:c.427C>T p.(Gln143Ter)	VUS	Depressed nasal bridge; Hypotonia; Moderate global developmental delay; Protruding tongue; Synophrys
**1032084**	Clinvar	NM_002742.3:c.445C>G (p.Leu149Val)	VUS	Congenital heart defects and ectodermal dysplasia
**982667**	Clinvar	NM_002742.3:c.496A>G (p.Met166Val)	VUS	Congenital heart defects and ectodermal dysplasia
**728781**	Clinvar	NM_002742.3:c.646C>G (p.Arg216Gly)	VUS	Not provided/ not specified, Premature ovarian failure
**PatB**	Jin et al. 2017 PMID: 28991257	NM_002742.3:c.727C>T p.(Arg243Ter)	Not specified	Ebstein malformation; L-TGA Usual coronary arteries in D-loop; Ventricular inversion
**PatA**	Jin et al. 2017 PMID: 28991257	NM_002742.3:c.813C>A p.(His271Gln)	Not specified	ASDII; PVS; Single ventricle; TS; VSD
**261674**	Decipher/Sifrim et al. 2016 PMID 27479907	NM_002742.3:c.896T>G (p.Leu299Trp)	Likely Pathogenic	AVSD; Attention deficit hyperactivity disorder; Chiari type I malformation; Microcephaly; Nystagmus; Specific learning disability
**803014**	Clinvar	NM_002742.3: c.1316G>A (p.Arg439Gln)	Likely pathogenic	Congenital heart defects and ectodermal dysplasia
**1032079**	Clinvar	NM_002742.3:c.1322G>A (p.Arg441Gln)	VUS	Congenital heart defects and ectodermal dysplasia
**931739**	Clinvar	NM_002742.3:c.1456G>A (p.Gly486Arg)	VUS	Congenital heart defects and ectodermal dysplasia
**Pat.A**	Alter et al. PMID 32817298	NM_002742.2: c.1774G>C p.(Gly592Arg)	Likely pathogenic	PVS, Thin, sparse hair, sparse eyebrows, deep set protruding ears, high palate, Premature loss of primary teeth, reduced number of permanent teeth, Short thumbs and short first toes, short 3rd and fourth metacarpals and metatarsals , Thin translucent skin, teleangiectasia since 14/15 years, flattened vertebral bodies with prominent pedicles, bilateral hip dysplasia

Sample/ Variant-ID	Source	Variant	Reported classification	Phenotype
**263568**	Decipher/Sifrim et al. 2016 PMID 27479907	NM_002742.3: c.1774G>C p.(Gly592Arg)	VUS	2-3 toe syndactyly; Abnormal cardiac septum morphology; Abnormality of prenatal development or birth; Brachycephaly; Deep plantar creases; Depressed nasal bridge; Finger syndactyly; Fragile nails; Frontal bossing; Generalized hypotonia; High anterior hairline; Hypoplasia of teeth; Melanocytic nevus; Scoliosis; Sparse scalp hair; Stridor; Thin skin
**268276**	Decipher/Sifrim et al. 2016 PMID 27479907	NM_002742.3: c.1774G>C p.(Gly592Arg)	Pathogenic	Bilateral conductive hearing impairment; Broad thumb; Delayed speech and language development; Dry skin; Premature loss of primary teeth; Pulmonic stenosis; Short digit; Sparse scalp hair
**Pat.B**	Alter et al. PMID 32817298	NM_002742.2: c.1808G>A p.(Arg603His)	VUS	Recurrent middle ear infections, bilateral cholesteatoma, PVS (haemodynamically irrelevant), sparce hair, frontal bossing, pectus excavatum, shortening of hands and feet, toe-syndactyly, telangiectasia
**Pat.A**	Alghaith et al. 2023 PMID: 36308391	NM_002742.3:c.1808G>T p.(Arg603Leu)	Likely Pathogenic	AVSD, AS, PS, PAPVR, sparse scalp hair, dysmorphic facial features, premature loss of primary teeth, small and widely spaced teeth, scoliosis, stabismus, nephrocalcinosis, medullary sponge kidney, bilateral papilledema
**413370**	Decipher	NM_002742.3:c.1808G>A p.(Arg603His)	VUS	AVSD; Cleft palate; Deep plantar creases; Duplicated collecting system; Intestinal malrotation; Intrauterine growth retardation; Low-set, posteriorly rotated ears; PDA; Prominent nasal tip; Pulmonary artery stenosis; Sandal gap
**203494**	Clinvar	NM_001330069.2: c.1876C>T (p.Arg626Ter)	VUS	Not provided/ not specified
**1032081**	Clinvar	NM_002742.3:c.1947T>G (p.Phe649Leu)	VUS	Congenital heart defects and ectodermal dysplasia
**161599**	Clinvar	NM_002742.3:c.1993G>A p.(Asp665Asn)	VUS	Malignant tumor of prostate
**1032082**	Clinvar	NM_002742.3:c.2068-11C>A p.(?)	VUS	Congenital heart defects and ectodermal dysplasia
**998171**	Clinvar	NM_002742.3:c.2219G>A (p.Arg740Gln)	VUS	Congenital heart defects and ectodermal dysplasia
**493141**	Clinvar	NM_002742.3:c.2280C>T (p.Tyr760=)	VUS	Not provided/ not specified
**Pat.A**	Sifrim et al. 2016 PMID 27479907	NM_002742.3:c.2288C>T (p.Ser786Phe)	VUS	Aortic coarctation, Bicuspid aortic valve

Sample/ Variant-ID	Source	Variant	Reported classification	Phenotype
**PatC**	Jin et al. 2017 PMID: 28991257	NM_002742.3:c.2380C>T p.(Gln794Ter)	Not specified	ASDII; PDA: PVS
**PatD**	Jin et al. 2017 PMID: 28991257	NM_002742.3:c.2739A>T p.(Ter913CysextTer21)	Not specified	Aberant right subclavian artery; Abnormal aortic arch; AS; BAV; DORV; VSD; LSVC to coronary sinus; SDS; Tubular hypoplasia of aorta and coarctation

AS, aortic valve stenosis; ASD, atrial septal defect; AVSD, atrioventricular septal defect; BAV, bicuspid aortic valve; DORV, double outlet right ventricle; LSVC, left superior vena cava; PAPVR, partial anomalous pulmonary venous return; PDA, patent ductus arteriosus; PVS, pulmonary valve stenosis; SDS, sudden death syndrome; TA, tricuspid valve atresia; TGA, transposition of the great arteries; TS, tricuspid stenosis; VSD, ventricular septal defect; VUS, variant of uncertain significance.

Table S2. Primers sequences used for genotyping and RT-qPCR assays. Bp, base pair; N/A, not applicable.

Gene	Primer sequence (5' to 3')	Length (bp)	Efficiency (%) / R^2
**Primers for genotyping**			
**Wild type**	Forward: GGCATGGCTGGACCTAATCT<br>Reverse: CACCCATGCCCTCAAGTATC	223	N/A
**Mutant**	Forward: GGCATGGCTGGACCTAATCT<br>Reverse: GCTTGACACTGGAAATGGAA	212	N/A
**Primers for RT-qPCR**			
**Pgk1**	Forward: GTCGTGATGAGGGTGGACTT<br>Reverse: AAGGACAACGGACTTGGCTC	126	108.9 / 0.99
**Rpl**	Forward: GCCGCTGGTGGTTGAAGATAA<br>Reverse: CGTCGGTTTCTCATTTTGCCC	150	109 / 0.99
**Prkd1**	Forward: ATGTATCCACCCAACCCGTG<br>Reverse: CCACCTGGAGTCATCGCTTT	213	99 / 0.98
**Prkd2**	Forward: GTGAGCCTTAGTGGCACGTT<br>Reverse: GTCAAGCCACGTCTGGTACT	219	99.75 / 0.98
**Prkd3**	Forward: CCACCAAATCCATGGCGAGA<br>Reverse: CCAGATTGTGCGTGTATGCG	235	86.19 / 0.99

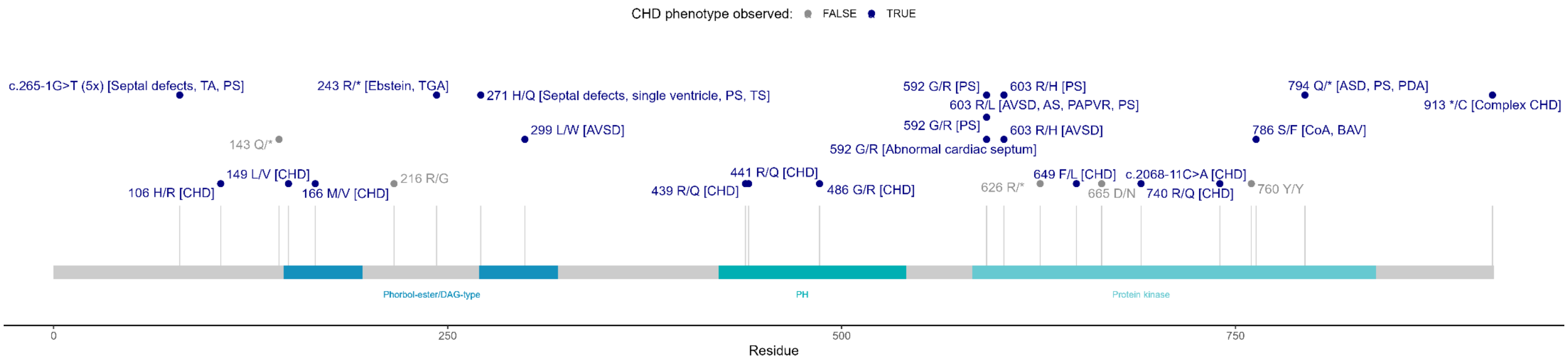

Figure S1. Figure S1. Plot of published PRKD1 variants. Distribution of variants as defined in Supplementary Table S1. Functional domains were marked based on Uniprot: Phorbol-ester/DAG-type, Phorbol esters/diacylglycerol binding domain (C1 domain); PH, pleckstrin homology domain. Dark blue variants were found in samples with reported CHD phenotype, variants depicted in grey were not reported in a CHD context. Indicated in the brackets is the reported CHD. ASD, atrial septal defect; AVSD, Atrioventricular septal defect; BAV, bicuspid aortic valve; CHD, not specified form of CHD; CoA, aortic coarcation; PAPVR, partial anomalous pulmonary venous return; PDA, patent ductus arteriosus; PS, pulmonary stenosis; TA, tricuspid valve atresia; TGA, transposition of the great arteries; TS, tricuspid valve stenosis; VSD, ventricular septal defect.

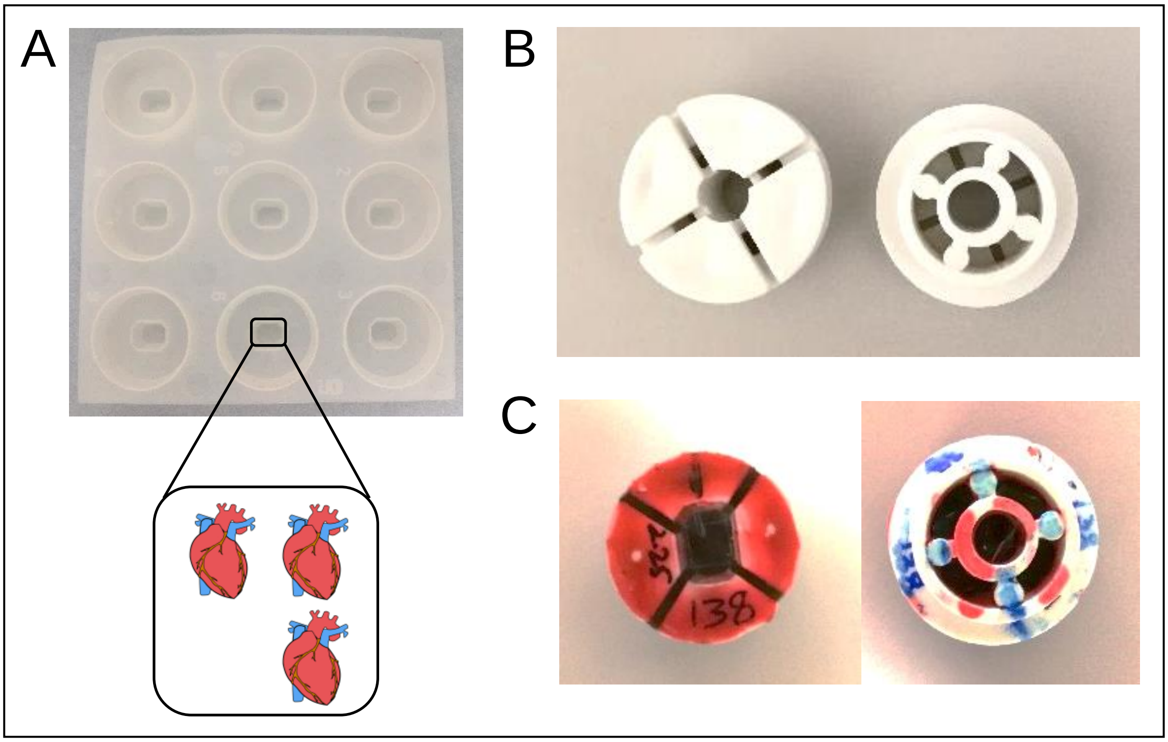

Figure S2. Embedding for HREM. A. Embedding mould and hearts placement inside the mould. **B.** Embedding chucks. **C.** Blocks after embedding.

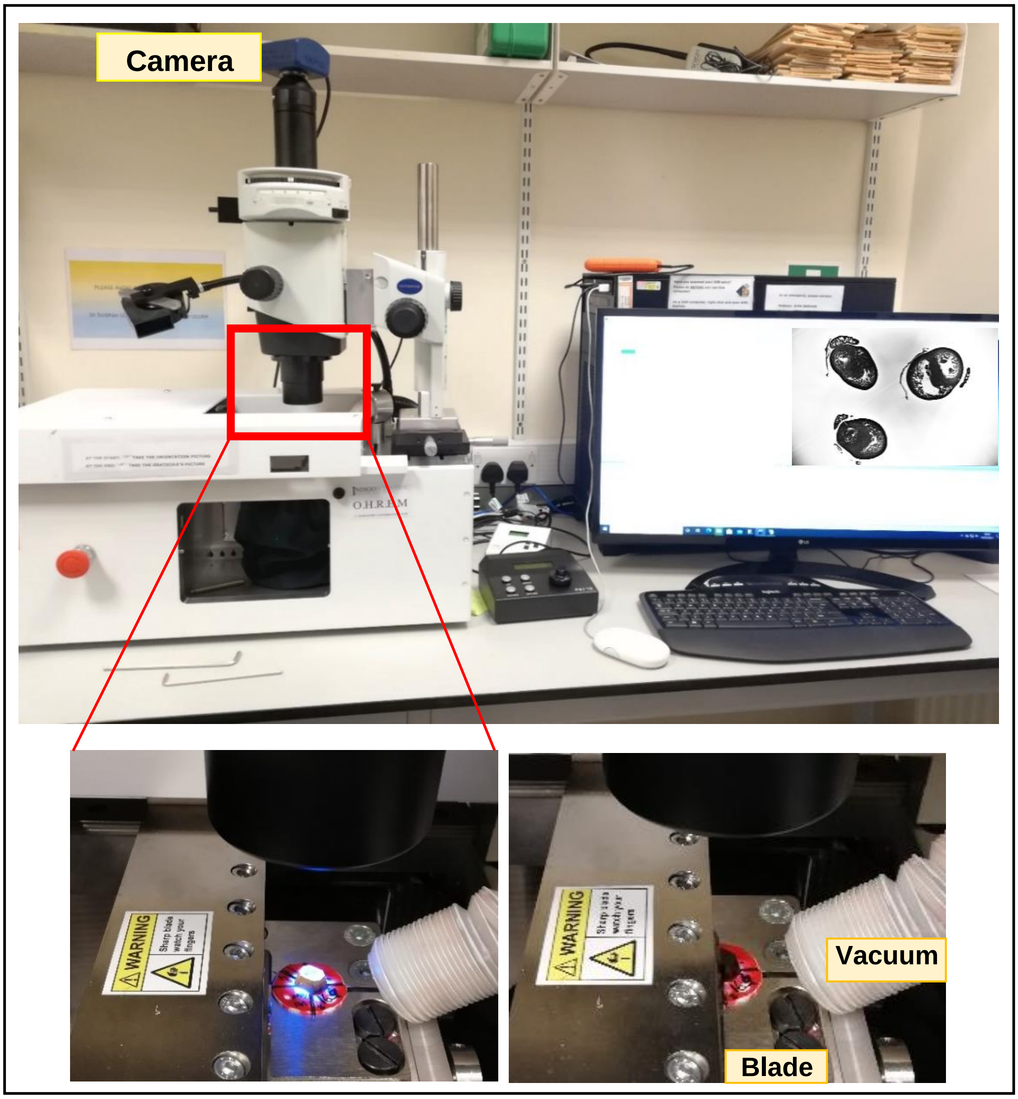

Figure S3. HREM set up. The HREM connected to the camera and the computer to capture images. A close-up on the area demarcated by the red square shows the block with an image being taken as can be seen on the computer screen. After taking the image of the block's surface, the blade cuts through the section, which then gets removed by the vacumn.

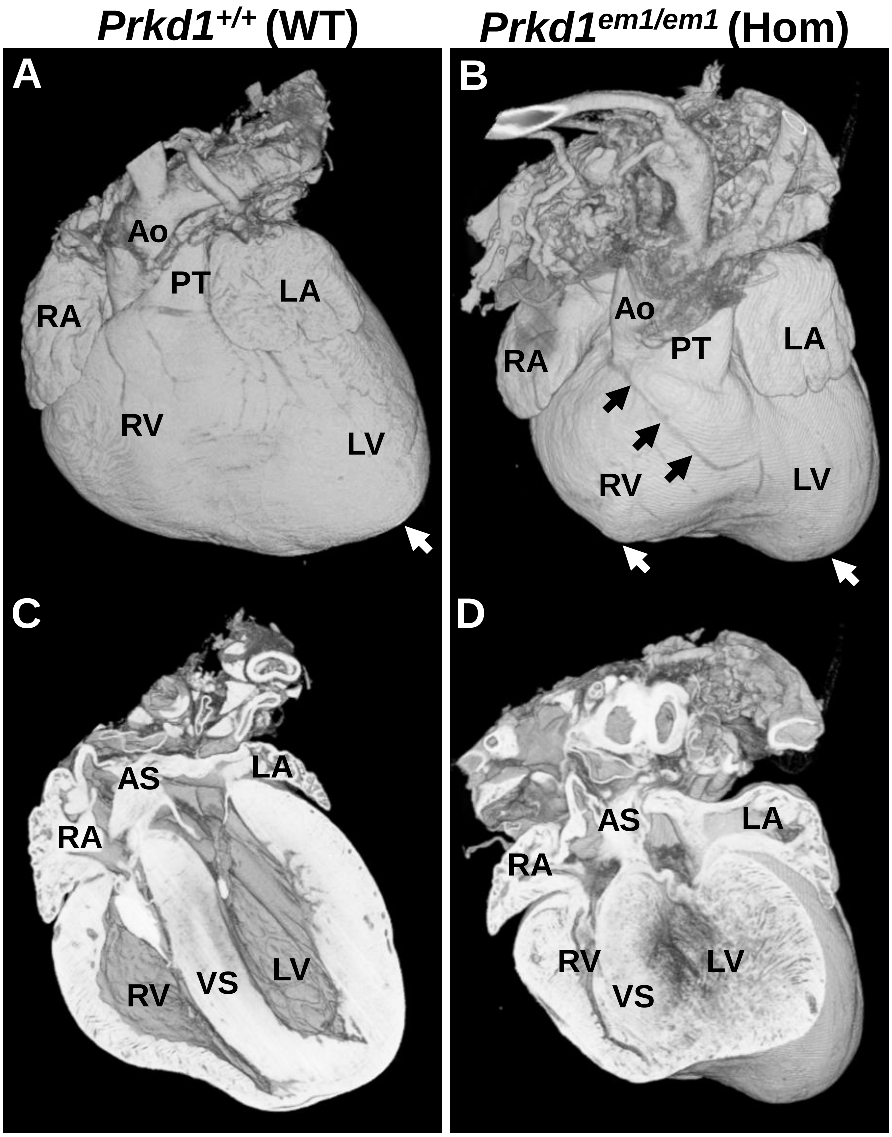

Figure S4. Comparison of *Prkd1*$^{+/+}$ and *Prkd1*$^{em1/em1}$ hearts at P7. A-B. Hearts rotated in a rightward direction in comparison to Figure 6, External view of *Prkd1*$^{em1/em1}$ (homozygous) heart showing prominent bifid apices (white arrows), in comparison to *Prkd1*$^{+/+}$ (WT) control where one apex is denoted. The anterior interventricular coronary artery can be seen crossing across the right ventricle (black arrows), instead of in the anterior interventricular sulcus. **C-D.** Four chamber view showing normal atrial and ventricular septation in the homozygous heart, comparable to WT control. Ao, aorta; AS, atrial septum; LA, left atrium; LV, left ventricle; PT, pulmonary trunk; RA, right atrium; RV, right ventricle; VS, ventricular septum; WT, wild type.
